# Enhancing Medical Image Classification with an Advanced Feature Selection Algorithm: A Novel Approach to Improving the Cuckoo Search Algorithm by Incorporating Caputo Fractional Order

**DOI:** 10.3390/diagnostics14111191

**Published:** 2024-06-05

**Authors:** Abduljlil Abduljlil Ali Abduljlil Habeb, Mundher Mohammed Taresh, Jintang Li, Zhan Gao, Ningbo Zhu

**Affiliations:** 1 College of Computer Science and Electronic Engineering, Hunan University, Changsha 410012, China; habebabduljlil@gmail.com (A.A.A.A.H.); jintangl@usc.edu (J.L.); quietwave@hnu.edu.cn (N.Z.); 2 College of Engineering and Information Technology, Taiz University, Taiz, Yemen; mundhertaresh@taiz.edu.ye; 3 Research Institute of Hunan University in Chongqing, Chongqing 400000, China

**Keywords:** glaucoma, cuckoo search, Caputo fractional order, feature selection

## Abstract

Glaucoma is a chronic eye condition that seriously impairs vision and requires early diagnosis and treatment. Automated detection techniques are essential for obtaining a timely diagnosis. In this paper, we propose a novel method for feature selection that integrates the cuckoo search algorithm with Caputo fractional order (CFO-CS) to enhance the performance of glaucoma classification. However, when using the infinite series, the Caputo definition has memory length truncation issues. Therefore, we suggest a fixed memory step and an adjustable term count for optimization. We conducted experiments integrating various feature extraction techniques, including histograms of oriented gradients (HOGs), local binary patterns (LBPs), and deep features from MobileNet and VGG19, to create a unified vector. We evaluate the informative features selected from the proposed method using the k-nearest neighbor. Furthermore, we use data augmentation to enhance the diversity and quantity of the training set. The proposed method enhances convergence speed and the attainment of optimal solutions during training. The results demonstrate superior performance on the test set, achieving 92.62% accuracy, 94.70% precision, 93.52% F1-Score, 92.98% specificity, 92.36% sensitivity, and 85.00% Matthew’s correlation coefficient. The results confirm the efficiency of the proposed method, rendering it a generalizable and applicable technique in ophthalmology.

## 1. Introduction

Glaucoma is characterized by damage to the optic nerve, which leads to retinal ganglion cell degeneration. This degeneration affects neurons with cell bodies in the inner retina. As a result, the optic nerve receives its axons, and the affected nerves may atrophy, leading to cupping of the optic disc and a decrease in visual acuity. Regular eye exams are crucial for the early detection and treatment of glaucoma [1]. The exact factors that lead to the development of glaucoma and the biological mechanisms that cause it are still unknown. Although there is currently no known cure for glaucoma, the primary goal is to lower intraocular pressure to control the disease and prevent further optic nerve damage [2]. Regular monitoring and adherence to treatment plans are critical for optimal glaucoma management [3].

Fundoscopy stands out as a reliable diagnostic tool for the comprehensive evaluation of the fundus image, as it is characterized by simplicity of use and low cost. Although key tests such as pupillary reflex response and visual acuity help diagnose glaucoma, manual fundus image analysis is still subjective and dependent on the examiner’s experience. This subjectivity introduces the inherent risk of human error and potential discrepancies in diagnoses [4,5]. Moreover, using diverse instruments to take fundus images might result in inconsistent acquisition, including unclear or oblique images. Therefore, developing glaucoma diagnosis systems that include image recognition and adapt to different camera qualities is crucial for patient treatment.

Machine learning methods have become increasingly used in the field of medical diagnostics, particularly in the classification of glaucoma [6,7,8,9,10,11,12]. These methods leverage features extracted from fundus images to aid in accurate diagnosis. Although these methods have the potential for diagnosing glaucoma in clinical practice, there are limitations to using machine learning methods for glaucoma classification. The performance of these models depends on the quality and diversity of the dataset used for training. Further studies are needed to validate the performance of these models on larger and more diverse datasets. Additionally, the use of machine learning methods for glaucoma classification requires careful consideration of ethical and legal issues related to data privacy and security. To address these limitations, feature selection methods such as feature selection can be used to identify the most relevant and informative features for glaucoma classification. This method can help improve the accuracy and efficiency of machine-learning models for glaucoma classification.

Feature selection (FS) techniques are pivotal in improving dataset quality, decreasing dimensionality, reducing training time, and mitigating overfitting. Various FS techniques have been proposed in the task of glaucoma classification [13,14,15,16,17]. Despite the availability of various FS techniques, metaheuristic algorithms (MAs) have gained traction for their adaptability and efficacy in optimizing FS processes. These algorithms, including the genetic algorithm (GA) [18], the slap swarm algorithm (SSA) [19], particle swarm optimization (PSO) and artificial bee colony (ABC) [20], the golden jackal optimization algorithm (GJO) [21], and the bacterial foraging optimization algorithm (BFO) [22,23], exhibit remarkable flexibility in addressing the complexities inherent in glaucoma classification tasks. As FS can be viewed as an optimization challenge, there are no single metaheuristic algorithms capable of addressing all the complexities involved. This statement is established by the No-Free-Lunch (NFL) theorem [24]. Consequently, it becomes crucial to continue exploring novel alternative metaheuristic algorithms.

Yang and Deb introduced the cuckoo search (CS) algorithm [25,26], drawing inspiration from the breeding behavior of cuckoo birds (CB) for global optimization. In recent years, the application of CS has grown, making it a compelling choice for tackling intricate optimization challenges [27,28]. Moreover, the choice of CS is driven by its effectiveness in handling intricate and high-dimensional datasets [29]. Despite its advantages, CS is prone to suboptimal solutions due to an inadequate balance between exploration and exploitation phases [30]. To get around this constraint, the fractional-order–cuckoo search (FO-CS) algorithm has emerged as a powerful tool for enhancing the performance of traditional CS [31]. However, the intuitive interpretability of fractional-order models remains a challenge, prompting exploration into alternative approaches such as Caputo fractional order.

This paper presents an innovative design that combines Caputo fractional order with the cuckoo search algorithm (CFO-CS), making use of its stability and convergence advantages. We solve the truncation problems related to the Caputo definition by using a fractional-order gradient approach that incorporates a fixed memory step and an adjustable term count. Our method uses non-structural feature selection methods like histogram of oriented gradients (HOGs), local binary pattern (LBP), and deep features from MobileNet and VGG19 to make it easier to show glaucoma fundus images completely. We use a k-nearest neighbors (k-NN) classifier to evaluate the effectiveness of CFO-CS feature selection (FS) on glaucoma fundus images, which is a commonly used technique in such tasks [32,33,34]. We evaluate the proposed framework’s effectiveness using various metrics, including accuracy, F1 score, precision, specificity, sensitivity, and the Matthew correlation coefficient. The simulation outcomes demonstrate the significant superiority of the proposed framework over state-of-the-art models for glaucoma detection. Our contributions lay the groundwork for advancing glaucoma diagnostic techniques, promising improved accuracy and efficacy in clinical settings. Here is a summary of the current study’s primary contributions:

1. We propose a novel feature selection based on the integration of the Caputo fractional order with the CS algorithm to enhance the performance of the glaucoma fundus image classification model.
2. We provide a new way to obtain features from images that combines several strong methods, such as the histogram of oriented gradients (HOGs), the local binary pattern (LBP), and deep features from MobileNet and VGG19. This comprehensive approach ensures a robust and nuanced analysis of glaucoma fundus images, providing clinicians with valuable insights for accurate diagnosis and treatment planning.
3. To thoroughly evaluate our proposed method, we use a comprehensive dataset of actual glaucoma fundus images. By testing our method with unseen data, we validate its practical value and potential for widespread use, demonstrating its efficacy and reliability in practical medical applications.
4. We conducted a thorough comparative analysis of our newly designed feature selection method against existing techniques and other methods commonly used for glaucoma detection. This comprehensive evaluation highlights the superiority and distinctiveness of our approach, showcasing its competitive edge and significance in advancing the field of glaucoma diagnosis.

We anticipate that the findings from this study will pave the way for a revolutionary shift in enhancing feature selection methods and discovering new approaches to enhance the diagnosis of eye conditions through fundus images. This advancement holds the potential to significantly improve patient care within the field of ophthalmology. To the best of our knowledge, this paper represents the first instance of integrating the Caputo fractional order to the cuckoo search algorithm for feature selection in glaucoma fundus image classification. Furthermore, these contributions signify the transformative impact of our research, offering innovative solutions to address the challenges associated with glaucoma diagnosis.

The following structure guides the subsequent sections of this paper: Section 2 explains the study’s approach. Subsequently, Section 3 provides detailed outcomes and thorough explanations. Section 4 offers a comprehensive analysis of the findings. Finally, the last section summarizes our findings and indicates potential avenues for future studies.

## 2. Materials and Methods

**Figure 1:**
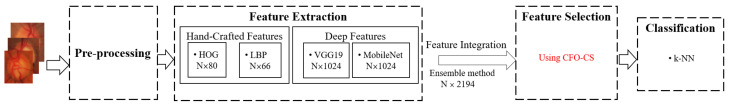
The outline of the proposed model.

As illustrated in Figure 1, we perform pre-processing, feature extraction, feature selection, and classification. During the pre-processing phase, images are resized to 224×224 and then converted to the RGB color space of 224×224×3. Thereafter, we extract shape-based, texture-based, and deep features using the histogram of oriented gradients (HOGs), local binary pattern (LBP), MobileNet, and VGG19 models. Afterward, we proceed to organize these extracted features and pass them through the CFO-CS feature selection method to determine the most relevant features. Subsequently, a k-NN classifier evaluates these features.

### 2.1. Dataset and Preprosessing

This study uses four extensively documented public databases: ORIGA, REFUGE, RIM-ONE DL, and ACRIMA, as explained in the existing literature [35]. The ORIGA dataset consists of 650 fundus images encompassing disc and cup segments [36]. The REFUGE dataset includes segmented fundus images and clinical glaucoma labels, totaling 1200 fundus images [37]. The recently introduced glaucoma dataset builds upon previous versions of RIM-ONE (v1, v2, and v3), comprising 485 fundus images [38]. The ACRIMA dataset, primarily captured from dilated eyes and centered on the optic disc, includes 705 fundus images [39]. As shown in Table 1, each dataset consists of two classes, with the quantity of each class denoted in bold.

**Figure 2:**
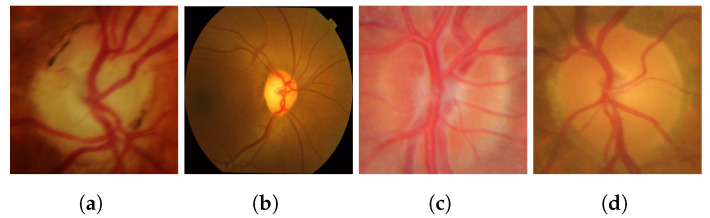
Sample images from the datasets: (**a**,**b**) glaucoma, (**c**,**d**) healthy images.

To provide a visual representation, the following images from the datasets are illustrated in Figure 2.

### 2.2. Image Feature Extraction Techniques

The histogram of oriented gradients (HOGs), the local binary pattern (LBP), VGG19, and MobileNet were used to extract features from images in this study. The HOG technique involves computing gradients, determining orientations (nine orientations were used in this study), and constructing histograms to reveal local gradient orientations within the image. It divides the image into 74×74 pixel cells and 1×1 pixel blocks, resulting in 80 features after aggregation. The LBP method is a robust local texture operator that conducts a binary comparison between outputs and neighboring pixels. With the implementation of eight sampling points and a radius of 1, the feature vector obtains a dimension of 66.

Furthermore, deep features are extracted using two pre-trained models, VGG19 and MobileNet, to enhance the performance. In the case of VGG19, we derive our feature vectors from the second fully connected layer (fc2), subsequently condensing them from an initial size of N×4096 to a more computationally efficient N×1024. On the other hand, the architecture of MobileNet, which uses separable convolutions, significantly reduces computational costs by an approximate 8–9-fold decrease in comparison to conventional methods. We extracted the features from the global average pooling layer, resulting in a feature set of N×1024. Moreover, we used a technique known as the ensemble approach to combine features from different feature extractions, including those from carefully constructed approaches and deep learning models. Consequently, we generated a unified set of 2194 features. We then select prominent features using CFO-CS and feed them into the k-NN classifier. We effectively leveraged each source’s distinct capabilities by using ensemble methods and stacking as a top-level model, resulting in a feature set that was more reliable and flexible.

### 2.3. Feature Selection (FS)

#### 2.3.1. Cuckoo Search Algorithm (CS)

The cuckoo search algorithm is a population-based meta-heuristic algorithm inspired by cuckoo brood parasitism, employing Lévy flights to enhance its performance. The fundamental ideas behind the CS approach are as follows:

1. Cuckoos lay eggs in randomly selected nests. 
2. Nests of the highest quality are inherited across generations.
3. Hosts have a predetermined number of nests, and the probability of a host encountering a foreign egg is denoted by $P_{a}$ in the range [0, 1]. In this scenario, the host bird can either discard the egg or abandon the nest.

The CS method uses Lévy flight and random walks to generate two populations of potential solutions. However, the random nature of the Lévy function leads to a significant diversity of individuals during exploration in search space. A switching parameter $P_{a}$ combines local and global random walks in CS. The global random walk uses the Lévy flight operation (λ) $∼u=k^{-\lambda },1<\lambda <3$ to move through the search space, i.e.,

(1) $$x_{j}^{\left(k+1\right)}=x_{j}^{\left(k\right)}+\beta ⊗\mathrm{Lévy}\left(\lambda \right).\;$$

Both $x_{p}^{k}$ and $x_{q}^{k}$ are solutions that are chosen at random by permutation in the local random walk. By using the variables, the current position $x_{j}^{\left(k\right)}$, step size β, step scaling factor *s*, Heaviside function H(u), probability of discovering a cuckoo egg $P_{a}$, the element-wise product of vectors ⊗ and *v*, which are drawn at random from a uniform distribution, the new position of the *j*th nest at the *k*th iteration is computed using (2). 

(2) $$x_{j}^{\left(k+1\right)}=x_{j}^{\left(k\right)}+\beta s⊗H\left(P_{a}-v\right)⊗H\left(x_{p}^{k}-x_{q}^{k}\right).\;$$

Algorithm 1 provides a summary of the primary procedures of the CS algorithm. The process consists of cuckoo identification, solution creation, assessment, nest replacement, desertion, and update. Based on the present location and the probability of a change, the stochastic (2) defines a random walk. To prevent becoming trapped in local optima and make sure the search region is sufficiently explored, it is critical to create fresh solutions using far-field randomization in CS.
**Algorithm 1** Cuckoo search via Lévy flightsGenerate initial population of *n* host nests $x_{j}$(j=0,1,⋯,n)**while** (k<MaxGeneration) or (stop criterion) **do**      Get a cuckoo randomly by Lévy flights using (1)      Evaluate its solution quality or objective value $f(x_{j})$       Choose a nest among *n* (say, *l*) randomly      **if** ($f\left(x_{j}\right)\;<f\left(x_{l}\right)$) **then**          Replace *l* by the new solution *j*      **end if**      A fraction ($P_{a}$) of worse nests are abandoned      New nests/solutions are built using (2)      Keep the best solutions (or nests with quality solutions)      Rank the solutions and find the current best      Pass the current best to the next generation**end while**

#### 2.3.2. Caputo Fractional Order with Cuckoo Search Algorithm (CFO-CS)

In CS, the step size is very important and has to be considered carefully when determining the suitable search area. Our method maintains these specified step size values from one generation to the next. However, this rigidity could potentially cause problems, such as the algorithm becoming stuck in local optima, thereby complicating the search for the optimal solution.

CS uses the Lévy flying method to allow cuckoo nests to travel through a combination of short and intermittent long-distance cooperative random searches. This setting is responsible for the significant and unexpected jumps observed in CS during the search process. However, this approach fails to thoroughly explore the surrounding region of each cuckoo nest, resulting in insufficient convergence accuracy and poor search robustness. A vector starting at the $x_{worst}$ point and ending at the $x_{best}$ point indicates the gradient direction, which corresponds to the direction of the maximum change. A function $f_{i,x}:R\to R$ is defined for i=1,…,d as $f(x+\left(y-x_{i}\right)e_{i})$, where $e_{i}$ is a vector in $R^{d}$ with a 1 in the *i*-th coordinate and 0’s elsewhere. Assuming a $R^{d}$ vector $c_{k}=c_{0},c_{1},\ldots ,c_{d}$, we may express the Caputo fractional order of *f* as

(3) ∇xαckCf(x(k))=DxαckCf(1,x)(x1),DxαckCf(2,x)(x2),…,DxαckCf(d,x)(xd).

Fractional calculus extends derivatives and integrals to non-integer orders, offering a precise tool for complex physical systems. Caputo’s definition, where the fractional derivative of a constant function is 0, is widely used in engineering problem-solving. The Caputo differential is a common expression used for this purpose. For a function *f* defined in [$c_{k}$,*x*], the fractional derivative to a real order α can be expressed using Caputo’s definition as:

(4) ∇xαckCf(x(k))=1Γ(r−α)∫ckx(x−τ)t−α−1f(t)(τ)dτ.

where $\Gamma \left(\alpha \right)=\int_{0}^{\infty }x^{\left(k)(\alpha -1\right)}e^{-x^{\left(k\right)}}dx$ is the gamma function, α∈[t−1,t], and *t* is a positive integer near α. In the discrete form, inspired by [40], Equation (4) could be formulated as

(5) ∇xαckCf(x(k))=∑v=t∞f(v)(x(k))Γ(v+1−α)(x−ck)(v−α).

For the updated rule to be replaced by (6), we need to truncate the higher terms v>M, where *M* is the size of the memory slot and converges to an extreme point 0<α<1.

(6) ∇xαckCf(x(k))=∑v=1Mf(v)(x(k))Γ(v+1−α)(x−ck)(v−α).

In this research, we use M≤4 terms to keep the execution time within an acceptable range since we are convinced that adding more memory terms increases the execution time. Furthermore, we incorporate the Caputo fractional given in (6) into (1) to enhance exploration in feasible regions, which yields

(7) xj(k+1)=xj(k)+βk⊗Lévy(λ)∇xαckCf(xbest(k)).

The average of the three solutions is used to determine $c_{k}$. The equation states that $x_{1,2,3}=x_{j}-r_{1,2,3}\left(x_{\mathrm{best}}-x_{j}^{\left(k\right)}\right)$, where $r_{1,2,3}$ represents uniformly distributed random values in the range [0,1]. This random number facilitates the exploration of new regions and prevents local minima stagnation. Given that $c_{k}$ is allocated to $x_{worst}$, in the same way, we enhance the exploitation around the best solution so far, which yields (8), by including the Caputo fractional provided in (6) into (2).

(8) xj(k+1)=xj(k)+βs⊗H(Pa−v)⊗∇xαckCf(xbest(k)).

#### 2.3.3. CFO-CS for Feature Selection

The CFO-CS feature selection begins by splitting the dataset into 70% training and 30% testing sets. It then uses a stochastic technique to generate *n* real-valued solutions. Each solution is created by randomly selecting values within predetermined dimension limits determined by the dataset features.

(9) $$x_{j}={\mathrm{lb}}_{j}+r_{1}\times \left({\mathrm{ub}}_{j}-{\mathrm{lb}}_{j}\right),\;$$

where the upper and lower bounds of the search domain *j* are denoted by ub and lb, respectively, and a random integer chosen from a uniform distribution is denoted by $r_{1}\in \left[0,1\right]$. Before we start updating, we calculate each solution’s fitness value. We transform the real value of each solution to binary, as shown in (10), to address the discrete nature of the FS issue.

(10) $$F_{j}=\left\{\begin{array}{cc} \;1 & \mathrm{if}\;x_{j}>0.5\\ \;0 & \mathrm{otherwise}\; \end{array}\right.,$$

The fitness value $f_{j}$ for each solution *j* is computed using Equation (11)

(11) $$f_{j}=\lambda \times \eta_{j}+\left(1-\lambda \right)\times \left(\frac{|F_{j}|}{\dim}\right),\;$$

where $F_{j}$ is a binary vector indicating selected features for the $j^{th}$ solution, $|F_{j}|$ denotes the number of selected features in the $j^{th}$ solution, dim is the total number of features, λ is a parameter balancing between performance and feature sparsity, and $\eta_{j}$ represents the performance measure of the $j^{th}$ solution using the selected features. The first term $\lambda \times \eta_{j}$ emphasizes the importance of performance, where λ controls the weight given to $\eta_{i}$, while the second term $\left(1-\lambda \right)\times \left(\frac{|F_{j}|}{\dim}\right)$ penalizes sparsity, encouraging the selection of more relevant features. By adjusting the parameter λ, the trade-off between performance and sparsity can be tuned to suit the specific requirements of the problem. This fitness evaluation strategy guides our feature selection algorithm toward identifying feature subsets that optimize both performance and sparsity. Subsequently, the solutions *x* are updated using the operations defined in Equations (7) and (8), as indicated in Algorithm 2.
**Algorithm 2** Pseudo code of CFO-CSObjective Function $f\left(x\right),x={\left(x_{1},...,x_{d}\right)}^{T}$ Generate initial population of *n* host nests $x_{j}$(j=0,1,⋯,n)Calculate the fitness value y−j**while** (*k* <MaxGeneration) or (stop criterion) **do**      Get a cuckoo randomly by Lévy flights using (7)      Evaluate its solution quality or objective value $f(x_{j})$       Choose a nest among *n* (say, *l*) randomly      **if** ($f\left(x_{j}\right)\;<f\left(x_{l}\right)$) **then**          Replace *l* by the new solution *j*      **end if**      A fraction ($P_{a}$) of worse nests are abandoned      New nests/solutions are built using (8)      Keep the best solutions (or nests with quality solutions)      Rank the solutions and find the current best      Pass the current best to the next generation**end while**

### 2.4. Evaluating Metrics

In this section, we analyze the performance of classifiers in classifying glaucoma fundus images using various evaluation metrics, primarily derived from the confusion matrix. The confusion matrix serves as a fundamental reference for assessing the performance of classifiers by categorizing predictions into four potential outcomes: false negative (FN), false positive (FP), true negative (TN), and true positive (TP). Both false negatives and false positives can have serious consequences in medical decision-making. False negatives can lead to missed diagnoses and a failure to treat patients who need care. False positives, on the other hand, can lead to unnecessary treatments, additional testing, and patient anxiety. Therefore, medical diagnostic models must have high true positive and true negative rates and low false positive and false negative rates.

To quantitatively evaluate classifier performance on the test set, we employ several key metrics: accuracy (Acc), F1 score, precision (Pre), specificity (Spc), sensitivity (Sen), and the Matthew correlation coefficient (Mcc). These metrics provide a comprehensive assessment of classifier performance and are defined as follows:

(12) $$\begin{array}{cc} \mathrm{Acc} & =\frac{TP+TN}{TP+TN+FP+FN} \end{array}$$

(13) $$\begin{array}{cc} \mathrm{Pre} & =\frac{TP}{TP+FP} \end{array}$$

(14) $$\begin{array}{cc} \mathrm{Spc} & =\frac{TN}{TN+FP} \end{array}$$

(15) $$\begin{array}{cc} \mathrm{Sen} & =\frac{TP}{FN+TP} \end{array}$$

(16) $$\begin{array}{cc} F1\;\mathrm{score} & =\frac{2\times \mathrm{Pre}\times \mathrm{Sen}}{\mathrm{Pre}+\mathrm{Sen}} \end{array}$$

(17) $$\begin{array}{cc} \mathrm{Mcc} & =\frac{\left(TP\times TN)-(FP\times FN\right)}{\sqrt{\left(TP+FP)(TP+FN)(TN+FP)(TN+FN\right)}} \end{array}$$

### 2.5. Experimental Setup

We use the ORIGA, REFUGE, and RIM-ONE DL datasets at a ratio of 70:30 during the training phase. For the testing phase, we specifically reserve the ACRIMA dataset as unseen data. We conducted this study using the Python programming language on a Tesla K80 GPU within the Google Colaboratory. The hardware setup includes an Intel© i7-core processor @3.6GHz, 16GB RAM, and operates on a 64-bit Windows 10 system. Additionally, this research integrates image augmentation algorithms with precise parameter values to effectively address the imbalanced issue, specifically targeting the minority class. These algorithms include geometric transformations such as flipping, rotation (±20 degrees), mirroring, zooming, shearing, and cropping (10% margin). We use the Keras ImageDataGenerator class for real-time image augmentation. This method guarantees that the transformed images preserve the diversity present in the original dataset, consequently minimizing the likelihood of overfitting by the selected classifiers.

The globally optimized solutions are derived through the parameters $P_{a}$, λ, and β as initially presented in the CS method. These parameters are instrumental in identifying locally superior solutions. The values of $P_{a}$, λ, and β are set as 0.25, 1.5, and 0.3, respectively. We set up the CS with a population size of 10 cuckoos (solutions) and 15 nests. Each trial involves running the CS for 100 generations (iterations). The values of the parameters used in this method are detailed in Table 2.

We implement the k-nearest neighbors (k-NN) model for image classification. The k-NN algorithm requires various input data, including the number of neighbors (*k*), a distance matrix, and a training dataset. We conducted a careful train–validation split to assess the model’s performance on a validation set and identify the optimal *k* value. The model completed multiple training grounds, with *k* values ranging from 1 to 10. We determined the optimal value for k=7, as shown in Table 3, where each evaluation metric’s highest value is bolded. The distance matrix was calculated using the Euclidean distance.

## 3. Results

**Figure 3:**
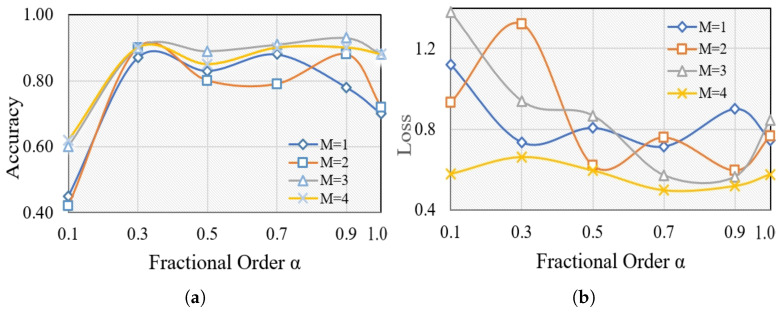
The average training (**a**) accuracy and (**b**) loss of different α for each *M*.

We conducted a series of experiments to validate the effectiveness of the proposed feature selection. Motivated by the long memory property of fractional-order derivatives, we enhance the fractional-order gradient method by introducing an adjustable number of terms (M=1,2,3,4). The main measures we employ to evaluate performance are the average training accuracy and loss. Each *M* is considered with varying fractional orders (α=0.1,0.3,0.5,0.7,0.9,1), where α=1 denotes training using CS (cuckoo search), while other values represent training with CFO-CS. We keep the number of iterations constant at 100 for each of the ten trials. Figure 3 illustrates the average training accuracy and loss for each *M* with varying α values, offering insights into performance variations under different fractional-order conditions.

The effectiveness of k-NN is evident for α≥0.7, as seen in the average training accuracy and loss. For α<0.5, the extremely large gamma function associated with fractional calculus results in low values. However, some undesirable outcomes occurred, notably at α=0.7 and 0.9, particularly with M=1 and 2. Generally, The average accuracy and loss improve as the fractional order and number of terms *M* increase, as shown in Figure 3. However, it appears that fractional order gradients influence the loss, resulting in repeated jumps. A higher variance typically indicates a more adaptable distribution, indicating that the fractional order gradient approach helps to optimize frequent and wide process jumps. This, in turn, increases the chances of avoiding local optimal points. Moreover, Figure 3 demonstrates that the optimal values for fractional-order gradient methods are achieved when M=4 and α=0.7. These values surpass those obtained using the integer-order gradient method. Therefore, we consistently maintained M=4 and α=0.7 throughout our experiments in this paper.

**Figure 4:**
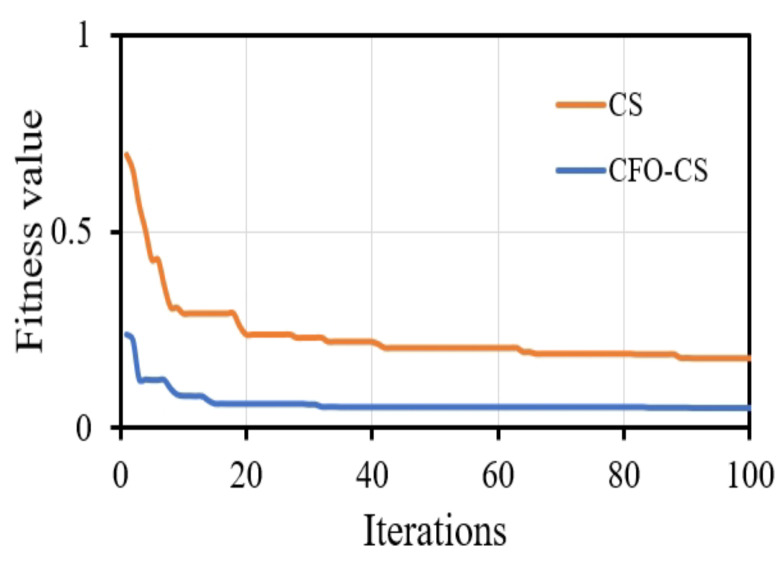
The convergence profile of using CFO-CS and CS for feature selection.

Visual representations of convergence speed help to understand the dynamic training process. It is important to recognize that presenting the convergence of classifiers without feature selection may not offer substantial insights into their training dynamics. This is because feature selection algorithms such as CS fundamentally change the optimization landscape by choosing a subset of informative features, thereby impacting the convergence behavior of classifiers. We chose to focus on visualizing convergence with CFO-CS because we want to draw attention to the dynamics of optimization in feature-selected scenarios. These are important in real-world situations where reducing the number of dimensions has a big effect on model performance. Figure 4 depicts the convergence behavior of the k-NN classifier using CFO-CS and CS for feature selection.

Figure 4 illustrates that CFO-CS exhibits faster convergence than CS in feature selection, highlighting its efficiency in swiftly adapting to selected relative features and enhancing performance. Conversely, CS may require more iterations for convergence, indicating relatively slower optimization progress. This indicates that in the optimization process, CFO-CS achieves stability and an optimal solution more quickly.

**Figure 5:**
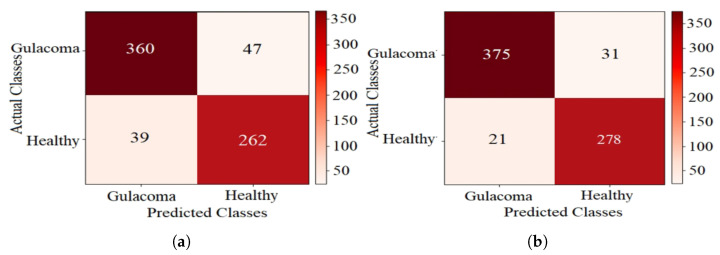
The confusion matrix of k-NN performance using CS for FS. (**a**) CS and (**b**) CFO-CS for FS.

The confusion matrices for k-NN using CFO-CS and CS feature selection are shown in Figure 5, providing a detailed breakdown of the test-set classification results. In Figure 5a, where CS is used for feature selection, the classifier accurately identified 360 glaucoma cases and 262 healthy cases. However, it exhibited confusion, with 39 healthy cases misclassified as glaucoma and 47 cases with glaucoma incorrectly labeled as healthy. Conversely, using CFO-CS for feature selection resulted in improved model performance on the test set, as shown in Figure 5b; 375 glaucoma cases were correctly classified. Nonetheless, there was confusion in 21 healthy cases misclassified as glaucoma and 31 cases with glaucoma mistakenly labeled as healthy.

Table 4 summarizes the performance metrics for CS and CFO-CS feature selection on the test set, highlighting the CFO-CS results in bold. The results show notable improvements with CFO-CS across all evaluated metrics. Specifically, CFO-CS demonstrates a significant increase in accuracy (92.62% vs. 89.36% with CS), precision (94.70% vs. 91.17% with CS), F1 Score (93.52% vs. 90.59% with CS), and Matthews correlation coefficient (85.00% vs. 78.36% with CS). These results highlight the effectiveness of CFO-CS in enhancing overall classification performance compared to the CS method.

Furthermore, we compared our approach to [41], which uses identical benchmark datasets without feature selection techniques. We also performed a comprehensive assessment in comparison to [42], where similar techniques were applied. For this comparison, we used the whale optimization algorithm (WOA) as a method to select features. We made sure to use the same settings and parameters as described in [42] to ensure a fair comparison. Table 5 demonstrates the numerical results of our comparison analysis. The obtained results confirm the efficacy of CFO-CS compared to methods used in both [41,42].

**Figure 6:**
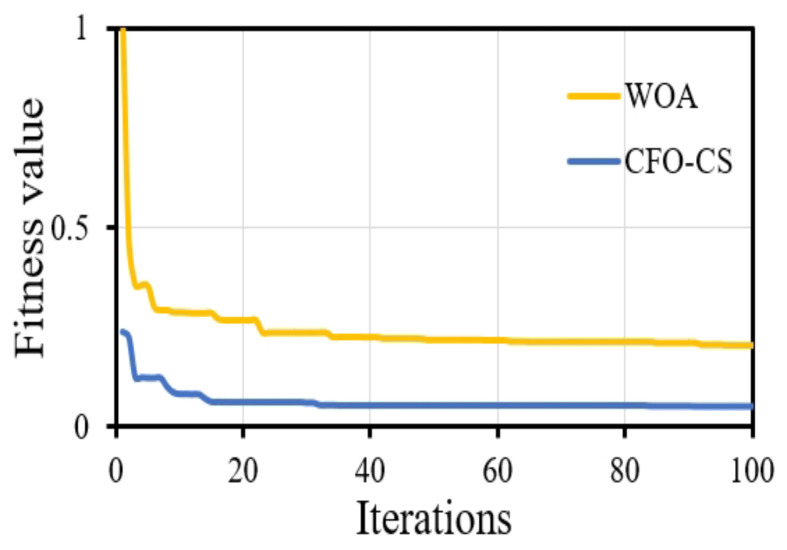
The convergence profile of using CFO-CS and WOA for feature selection.

Furthermore, CFO-CS outperforms the approach given in [42] in convergence behavior, indicating its effectiveness in identifying optimum solutions. Figure 6 shows the convergence diagram, which reveals that using CFO-CS as a feature selection technique leads to improved convergence dynamics.

## 4. Discussion

This work explores the effectiveness of Caputo’s definition of fractional-order methods in improving feature selection methods and enhancing the performance of k-NN classifiers for glaucoma diagnosis. By adjusting the number of terms (M=1,2,3,4) and exploring different fractional orders (α≤1), we evaluated the training average accuracy and loss using CFO-CS. Figure 3 revealed that k-NN performs well at α≥0.7 with higher values of *M*. Furthermore, it highlights the superiority of fractional-order gradient methods, particularly when M=4 and α=0.7, outperforming integer-order gradient methods in terms of average training accuracy and loss. These results indicate promising opportunities for leveraging fractional-order gradient methods to enhance machine learning algorithms for disease diagnosis applications.

Convergence speed is an essential component in gaining an understanding of the training dynamics of the classifier. As depicted in Figure 4, k-NN exhibits significantly faster convergence when using CFO-CS compared to CS for feature selection. The fast convergence observed in the convergence diagram indicates that an optimal solution is efficiently reached during the optimization process, which indicates a more stable and robust performance. On the contrary, using CS to select features requires more iterations to reach the optimal solution.

Figure 5 shows the confusion matrices, which give us a closer look at how well k-NN performs with different feature selection methods. In the context of disease diagnosis, the true positive (TP) and true negative (TN) rates are of paramount importance. These parameters directly reflect the model’s ability to correctly identify positive and negative cases, respectively. In Figure 5a, the use of CS for feature selection results in 360 true positives (TPs) and 262 true negatives (TNs). However, the application of CFO-CS for feature selection further enhances these metrics, yielding 375 TPs and 278 TNs, as shown in Figure 5b. This improvement highlights the effectiveness of CFO-CS in improving the accuracy of the k-NN classifier, particularly in distinguishing between glaucoma and healthy classes.

Furthermore, Table 4 compares the performance of the k-NN classifier in classifying the test dataset when using CFO-CS and CS for feature selection. The results obtained demonstrate significant enhancements across all evaluation metrics with the implementation of CFO-CS feature selection. These improvements confirm the effectiveness of CFO-CS in enhancing the predictive accuracy and overall robustness of classifiers employed in glaucoma diagnosis.

To further validate the effectiveness of our proposed method, we conducted a comparison with existing methods outlined in the literature, as shown in Table 5. Interestingly, our method works better than similar ones that use the whale optimization algorithm (WOA) for feature selection [42], which uses the same dataset but does not use any feature selection techniques [41]. This comparison serves to further validate the superiority of our method for enhancing performance.

To further validate the efficacy of CFO-CS, we conducted a comparative analysis with existing approaches documented in the literature, as summarized in Table 5. Notably, our method exhibits superior performance compared to similar techniques that utilize the whale optimization algorithm (WOA) for feature selection [42], which employs the same dataset without any feature selection methods [41]. This comparative evaluation further validates the superiority of our approach in enhancing overall performance, thereby underscoring its potential as a robust and effective strategy for optimizing classification accuracy and reliability.

The adaptability of our method to various feature extraction scenarios, coupled with the robust feature selection capabilities of CFO-CS, positions our approach as a promising tool for glaucoma diagnosis. The consistent superiority of the k-NN classifier and the efficiency of CFO-CS as a feature selection method underscore the strengths of our methodology. However, while our approach has demonstrated significant improvements in glaucoma classification, there is still room for further enhancement. Techniques such as image enhancement and noise reduction can potentially improve the quality of input data, thereby contributing to more accurate diagnoses. Additionally, it is important to note that CFO-CS can be sensitive to parameter settings, which must be carefully tuned to achieve optimal performance. The computational overhead associated with feature selection, especially in scenarios involving the integration of concatenated features, may pose challenges for real-time applications. Despite these considerations, our method has shown promising results, indicating its potential as a valuable tool in the field of glaucoma diagnosis. Moving forward, it will be essential to address these challenges through further refinement and adaptation to specific clinical contexts, ensuring the method’s effectiveness and reliability in real-world applications.

## 5. Conclusions and Future Work

The accurate detection of glaucoma using fundus images poses challenges due to image acquisition heterogeneity, including the capture of blurred images or images from different angles. To tackle these challenges, we introduced a novel method named CFO-CS, which combines the Caputo definition of fractional-order methods with the cuckoo search algorithm to enhance feature selection. We applied the data augmentation method to increase the number of training datasets. Subsequently, we extracted shape-based and texture features using the histogram of oriented gradients (HOGs) and local binary pattern (LBP) and obtained deep features using MobileNet and VGG19 from fundus images. These features were concatenated, resulting in a dimension of N×2194 before being supplied to the k-NN classifier. The performance of our proposed classification model was evaluated on challenging glaucoma datasets, achieving an accuracy of 92.62%, precision of 94.70%, F1-Score of 93.52%, specificity of 92.98%, sensitivity of 92.36%, and Matthew’s correlation coefficient of 85.00%. A comparison with the most recently published work confirmed that our method yielded superior results. Overall, our study sheds light on the crucial role of feature selection in enhancing the performance of machine learning classifiers, particularly in the field of medical image analysis focused on glaucoma classification. Future research could explore our method’s performance with larger datasets and address challenges such as improving image quality and reducing noise. Additionally, improvements could involve using advanced deep-learning models or combining different methods for better glaucoma classification.

## Figures and Tables

**Table 1:** Number of images for each class in the datasets.

Dataset	Glaucoma	Healthy
ORIGA	168	482
REFUGE	120	1080
RIM-ONE DL	172	313
ACRIMA	396	309
**Total**	856	2184

**Table 2:** The parameters used in this study for feature selection.

Parameters	Value
Population Size (n)	10
Cuckoo Eggs (nests)	15
$P_{a}$ (probability)	0.25
β	0.3
Iteration	100
λ	1.5

**Table 3:** The comparison of performance metrics (%).

*k*	Acc	Pre	F1 Score	Spe	Sen	Mcc
1	87.5	85.4	89.0	86.7	86.0	75.0
2	87.2	85.0	88.5	86.2	85.5	76.0
3	89.0	87.1	90.2	88.0	87.5	77.0
4	89.5	87.6	90.7	88.5	88.0	77.5
5	90.0	88.0	91.0	89.0	88.5	78.0
6	90.5	88.5	91.5	89.5	89.0	78.5
7	**91.0**	**89.0**	**92.0**	**90.0**	**89.5**	**79.0**
8	90.8	88.8	91.8	89.8	89.2	78.8
9	90.6	88.6	91.6	89.6	89.0	78.6
10	90.4	88.4	91.4	89.4	88.8	78.4

**Table 4:** The comparison of performance metrics (%).

Feature Selection	Acc	Pre	F1 Score	Spe	Sen	Mcc
CS	89.36	91.17	90.59	88.49	90.02	78.36
CFO-CS	**92.62**	**94.70**	**93.52**	**92.98**	**92.36**	**85.00**

**Table 5:** The comparison of performance metrics (%).

Feature Selection	Acc	Pre	F1 Score	Spe	Sen	Mcc
Method used in [41]	90.51	92.98	91.72	90.54	90.59	80.66
Method used in [42]	90.53	93.23	91.73	90.88	90.29	80.27
CFO-CS	**92.62**	**94.70**	**93.52**	**92.98**	**92.36**	**85.00**

## Data Availability

No new data were created.
